# Association between Body Image Flexibility and Intermittent Fasting in Chinese Medical Students: A Cross-Sectional Study

**DOI:** 10.3390/nu15194273

**Published:** 2023-10-06

**Authors:** Xinji Shi, Yibo Wu, Jie Yuan, Xue Wang, Chaowei Guo, Shuang Zang

**Affiliations:** 1 Department of Community Nursing, School of Nursing, China Medical University, Shenyang 110122, China; xjshi@cmu.edu.cn (X.S.); 13909822541@163.com (X.W.); gcw18830901603@126.com (C.G.); 2 School of Public Health, Peking University, Beijing 100191, China; bjmuwuyibo@outlook.com; 3 Jitang College of North China University of Science and Technology, Tangshan 063000, China; tsphyj@126.com

**Keywords:** body image flexibility, intermittent fasting, medical students, China

## Abstract

Unhealthy dietary behaviors and body dissatisfaction are becoming increasingly common among college students. Understanding the association between body image flexibility and intermittent fasting is particularly meaningful, especially for medical college students. This study aimed to investigate the association between body image flexibility and intermittent fasting among medical students. We conducted a cross-sectional study with 5138 medical college students at Jitang College of North China University of Science and Technology. Univariate and multivariate logistic regression were used to evaluate the association between body image flexibility and intermittent fasting. Subgroup analysis and interaction tests were further used to examine the possible interaction between body image flexibility and intermittent fasting. In this study, 1329 (25.87%) students had intermittent fasting behavior. After adjustment for confounding factors, there was a negative association between body image flexibility and intermittent fasting (OR = 0.94, 95%CI = 0.93 to 0.95, *p* < 0.001). A significant interaction between body image flexibility and intermittent fasting was found in gender, academic year, major, and monthly living expenses (*p* for interaction < 0.05). E-value analysis suggested there was unlikely to be an unmeasured confounding. This association could contribute to the establishment of personalized health intervention strategies and provide recommendations for promoting the physical and mental health of medical students.

## 1. Introduction

In recent years, body image flexibility has received widespread attention [1,2]. The definition of body image flexibility is the capacity to experience and accept unwanted thoughts and feelings about one’s body, enabling individuals to engage in actions consistent with their values despite concerns about body size, weight, or shape [3]. Thus, individuals with greater body image flexibility generally have a more accepting attitude toward their body shape and size and are less likely to display distress or reactivity in response to changes in weight. Conversely, those with lower body image flexibility are typically more distressed and anxious about their body image. Several studies have indicated that individuals with higher levels of body image flexibility are more likely to engage in positive health behaviors [4,5]. Furthermore, body image flexibility is closely associated with positive body image cognition [6] and mental health [7].

Intermittent fasting is becoming increasingly popular as a dietary strategy for weight management and health benefits [8]. Currently, research on intermittent fasting primarily focuses on its effects on weight loss and overall physical health [9,10,11,12].

Studies have demonstrated a correlation between diminished body image flexibility and eating disorders [1,13]. Eating disorders include dieting, fasting, etc. [14]. Although intermittent fasting and body image flexibility have garnered increasing attention in the field of health [6,15], the relationship between intermittent fasting and body image flexibility has received limited research attention to date. Particularly, there exists a scarcity of research investigating the association between intermittent fasting and body image flexibility among medical students. 

Understanding the association between body image flexibility and intermittent fasting is particularly interesting in the context of medical students. Medical students frequently encounter unique challenges, including high academic demands, rigorous coursework, and pressure related to clinical training [16,17]. Furthermore, medical education emphasizes the importance of understanding and promoting healthy lifestyles, such as professional nutrition education [18]. The characteristics of a medical students’ profession lead them to have a heightened focus on physical health and body image. As future healthcare practitioners, they need to interact with and care for patients, which requires them to possess a positive body image and health consciousness to effectively convey health information and set examples. Moreover, based on the different definitions of “body shape” and “beauty” in Chinese culture compared to the West [19], research on body image flexibility and intermittent fasting among Chinese medical students can help to better understand the manifestations of these phenomena. Therefore, studying the relationship between body image flexibility and intermittent fasting in medical students can offer insightful information about mental health and dietary behavior, serve as a foundation for creating individualized health interventions, and provide suggestions for future research.

The specific research questions (RQs) and hypotheses (Hs) in this study were as follows:

**RQ1:**  *Is there an association between body image flexibility and intermittent fasting among medical students?*

**H1:**  *There is a significant association between body image flexibility and intermittent fasting among medical students.*

**RQ2:**  *Are there other factors that influence the association between body image flexibility and intermittent fasting among medical students?*

**H2:**  *There are additional factors that influence the association between body image flexibility and intermittent fasting among medical students.*

## 2. Method

### 2.1. Study Design and Participants

The current cross-sectional study was conducted at Jitang College of North China University of Science and Technology (Tangshan, Hebei, China). This study used a single-centered cluster sampling method. The inclusion criteria were: (1) undergraduate students currently studying at the university; (2) volunteer to participate in this study; and (3) not in other similar studies. From November to December 2022, data were collected using a self-administered questionnaire. The questionnaire was distributed by trained investigators using WeChat (a social platform in China, with over one billion users) based on Questionnaire Star (a free online questionnaire survey program). In addition, the students were informed of the purpose of the study and related considerations. Informed consent was obtained from all participants, and all data were anonymized. 

This study was conducted under the Declaration of Helsinki and the Measures for Ethical Review of Biomedical Research Involving Human Beings [20]. Ethical approval was obtained through the Ethics Review Committee of Jitang College of North China University of Science and Technology (JTXY-2022-002).

First, this study included 5154 medical students. Then, 16 participants were excluded due to the missing values of body image flexibility and intermittent fasting. Finally, a total of 5138 participants were included in this study.

### 2.2. Measures

#### 2.2.1. Intermittent Fasting

In this study, intermittent fasting was defined in two ways: one as daily time-restricted fasting, with a shortened eating window of 6–8 h per day; the other as 5:2 fasting, which means that individuals only eat one medium-sized meal a day, and the calorie restriction is usually two days a week [21]. Participants were asked whether they had engaged in intermittent fasting behavior in the past year. Those who answered “yes” were identified as having intermittent fasting behavior, and those who answered “no” were considered not to have intermittent fasting behaviors.

#### 2.2.2. Body Image Flexibility

The Body Image-Acceptance and Action Questionnaire-5 (BI-AAQ-5) [22] was used to evaluate participants’ body image flexibility. The BI-AAQ-5 was an abbreviated version of the original version [3] with comparable performance [22,23]. It contained 5 items rated on a 7-point Likert-type scale (1 = never true to 7 = always true). All items (e.g., “Concern about weight makes it difficult for me to live a life that I consider worthwhile”) were reverse scored. A total score was calculated as the sum of all items (range: 5–35). Higher scores indicated higher levels of body image flexibility. In this study, Cronbach’s α for the BI-AAQ-5 was 0.94.

#### 2.2.3. Covariates

Based on prior studies [24,25,26,27,28,29,30], we identified several potential confounding variables, including age, body mass index (BMI), social media usage, family health, gender (limited to gender variable, without collecting sex information), ethnicity, academic year, major, hukou, place of residence, monthly living expenses, and love experience. 

##### Demographic Variables

Age, gender, academic year, and major were self-reported. The BMI was calculated based on self-reported height and weight. Ethnicity was classified into Han and minority. Hukou was divided into non-agricultural and agricultural. Place of residence was dichotomized into urban or rural. Monthly living expenses were a three-category variable: ≤800, 801–1500, and >1500 Chinese Yuan (CNY). Participants were asked, “What are your current monthly living expenses (for sophomores and seniors, choose the average for the past academic year; and for freshmen, choose the average for the past three months)?” Participants determined the variables of their love experience by answering the question “Are you in a relationship?”. And love experience was coded as a three-category variable: never been in love, have been in love, and are in love. 

##### Social Media Usage

Social media usage was measured with the 9-item Identity Bubble Reinforcement Scale [31]. Responses ranged from 1 (does not describe me at all) to 10 (describes me completely). The total score was the sum of all 9 items (range: 9–90). Higher scores reflected higher involvement in social media usage. In this study, Cronbach’s α of the 9-item Identity Bubble Reinforcement Scale was 0.93.

##### Family Health

The Short Form of the Family Health Scale (FHS-SF) [32] was a 10-item scale to measure family health. Responses to each item were rated on a 5-point Likert scale ranging from 1 (strongly disagree) to 5 (strongly agree). The sixth, ninth, and tenth items were scored in reverse (e.g., “In our family, we do not trust doctors and other health professionals”). A total score was the sum of all 10 items (range: 10–50). Higher scores reflected a higher family health degree. In this study, Cronbach’s α for the FHS-SF was 0.83.

### 2.3. Statistical Analysis

First, the data were assessed for normal distribution by the Kolmogorow–Smirnov test and Q-Q plots. Normally distributed continuous variables were presented as mean and standard deviation, and those that did not conform to a normal distribution were expressed as the median and interquartile range (IQR). Categorical variables were expressed as numbers and percentages. Participant characteristics were compared between groups using the *t*-test, Mann–Whitney U test, and Chi-square test. Second, a univariate logistic regression analysis was performed to identify factors associated with intermittent fasting. Multivariable binary logistic regression was used to explore the association between body image flexibility and intermittent fasting. Three models were used to adjust for potential confounders. Model I was unadjusted. Model II adjusted for age and gender. And Model III further adjusted variables with *p* < 0.05 in univariate analysis (i.e., age, BMI, social media usage, family health, gender, academic year, major, monthly living expenses, love experience). The results of the binary logistic regression models were shown as odds ratios (OR) with 95% confidence intervals (CI). Finally, we conducted a subgroup analysis and interaction test to explore the association between body image flexibility and intermittent fasting among different subgroups. To confirm the stability of the results, we calculated the E-value to assess potential unmeasured confounding [33]. 

Two-sided *p* < 0.05 was considered statistically significant. All statistical analyses were conducted using SPSS25.0 software (IBM Corp., New York, NY, USA) and R statistical package 4.2.0 (R Foundation for Statistical Computing, Vienna, Austria).

## 3. Result

### 3.1. Characteristics of Participants

A total of 5138 participants were included in this study. The age was 20.54 ± 1.62 years, and the median BMI was 22.23 kg/m^2^. The body image flexibility score was 25.24 ± 7.21, and 1329 (25.87%) participants had intermittent fasting behavior. The characteristics of participants divided by the median body imagery flexibility score were shown in Table 1. There were statistically significant differences in age, BMI, social media usage, family health, academic year, major, and intermittent fasting between the two groups (all *p* < 0.05). However, there were no statistically significant differences in gender, ethnicity, hukou, place of residence, monthly living expenses, and love experience (all *p* > 0.05).

### 3.2. Univariate Analysis Related to Intermittent Fasting

To screen potential risk factors related to intermittent fasting, we performed univariate binary logistic regression. Univariate analysis showed that body image flexibility, age, BMI, social media usage, family health, academic year, major, monthly living expenses, and love experience were associated with intermittent fasting (*p* < 0.05) (Table S1).

### 3.3. Association between Body Image Flexibility and Intermittent Fasting 

Binary logistic regression was employed for multivariate analysis. Statistically significant factors in the univariate logistic regression analysis were included in the multivariate logistic regression analysis. In addition, based on previous studies [34], we also adjusted for gender as a potential confounding factor. In the unadjusted model, body image flexibility was negatively associated with intermittent fasting. For each unit increase in body imagery flexibility score, the likelihood of intermittent fasting decreased by 7.23% (OR = 0.93, 95%CI = 0.92 to 0.94, *p* < 0.001). After adjusting for age and gender, the association between body image flexibility and intermittent fasting remained robust. Further adjusting for BMI, social media usage, family health, academic year, major, monthly living expenses, and love experience, the result was still statistically significant. The probability of intermittent fasting decreased by 5.88% for every unit increase in body image flexibility scores (OR = 0.94, 95%CI = 0.93 to 0.95, *p* < 0.001) (Table 2).

### 3.4. Subgroup and Interaction Analyses

**Figure 1:**
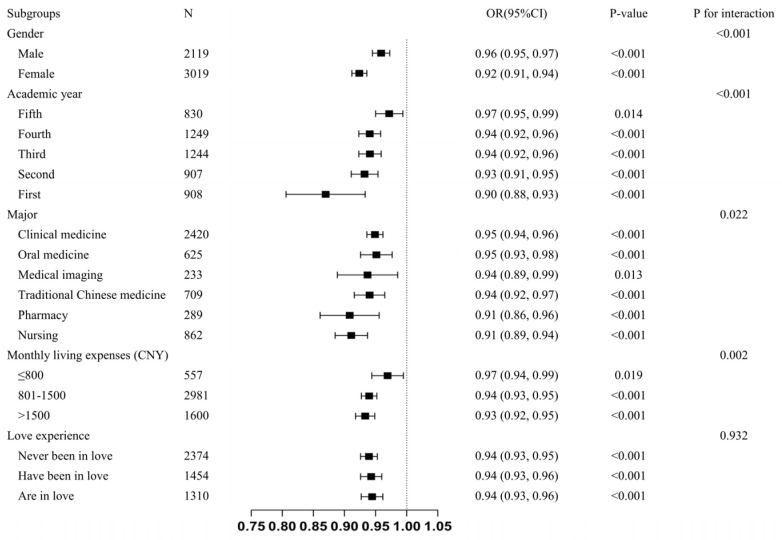
Association between body image flexibility and intermittent fasting in different subgroups. Note: The confounding variables (age, BMI, social media usage, family health, gender, academic year, major, monthly living expenses, and love experience) were adjusted for each stratification, except the stratification factor itself. BMI indicates body mass index. CNY indicates the Chinese Yuan. OR: odds ratio. 95%CI: 95% confidence interval.

To explore the role of the covariables in the association between body image flexibility and intermittent fasting, the subgroup analysis was conducted after stratifying the participants by gender, academic year, major, monthly living expenses, and love experience. The results of the subgroup analysis were shown in Figure 1, and regardless of subgroup, body imagery flexibility was negatively associated with intermittent fasting. In addition, we found a significant interaction between body image flexibility and intermittent fasting in gender, academic year, major, and monthly living expenses (*p* for interaction <0.05). However, the interaction was not significant in the love experience group (*p* for interaction >0.05). The negative association between body image flexibility and intermittent fasting was stronger among females (OR = 0.92, 95%CI = 0.91 to 0.94, *p* < 0.001), first year of college (OR = 0.90, 95%CI = 0.88 to 0.93, *p* < 0.001), pharmacy major (OR = 0.91, 95%CI = 0.86 to 0.96, *p* < 0.001), nursing major (OR = 0.91, 95%CI = 0.89 to 0.94, *p* < 0.001), and those with monthly living expenses >1500 CNY (OR = 0.93, 95%CI = 0.92 to 0.95, *p* < 0.001).

### 3.5. Sensitivity Analyses

In sensitivity analyses, the E-value was used to assess the degree of unmeasured confounding. The E-value (1.32) indicated that there was unlikely to be an unmeasured confounding affecting the association between body image flexibility and intermittent fasting. 

## 4. Discussion

In this study, we found a significant association between body image flexibility and intermittent fasting among Chinese medical students. This association was stronger in female students, those majoring in pharmacy and nursing, first-year students, and those with higher monthly living expenses. 

There was a negative association between body image flexibility and intermittent fasting among Chinese medical students, and a higher level of body image flexibility was associated with a lower likelihood of engaging in intermittent fasting behaviors. In this study, the overall BMI level of medical students who experienced intermittent fasting behavior was at a healthy level according to the World Health Organization (WHO) standard [35]. To explain the observed negative association between body image flexibility and intermittent fasting, the role of psychosocial factors needs to be considered. For example, society’s emphasis on “thin beauty” standards may lead to increased dissatisfaction with one’s body image [36], causing individuals to attempt intermittent fasting even at a healthy body size to look more aesthetically pleasing. Within the university environment, academic stress, social pressures, and self-identity issues may play a role in shaping students’ body image and dietary behaviors [26,37]. In this contextual backdrop, body image flexibility may reflect individuals’ different ways of coping with these pressures and factors. On the other hand, the adoption of intermittent fasting behaviors may be motivated by a multitude of factors, including concerns regarding weight management and overall physical well-being [10]. These motivations may interact with individuals’ body image, further influencing their dietary choices. The findings of this study offered new insights into enhancing the well-being of college students. College life is often accompanied by multiple stress factors, and cultivating body image flexibility may serve as a protective factor in preventing the adoption of extreme dietary behaviors, which can entail health risks and psychological burdens [38,39]. A deeper understanding of the mechanisms and long-term impacts of this association is crucial for the development of targeted intervention measures and support strategies, such as implementing psychological health support, promoting the formation of positive body image cognition, healthy eating education, and social support, to assist students in establishing healthy body images and dietary habits, thereby improving the physical and mental health of college students. Future research should consider intervention measures and health education programs to reduce unhealthy eating habits and promote positive body image.

In this study, we found a stronger negative association between female body image flexibility and intermittent fasting among medical college students. This gender difference was consistent with previous studies [40,41]. Firstly, unlike males, females were significantly impacted by changes in hormone levels during the menstrual cycle, which regulated dietary consumption as well as metabolic and physiological conditions [42]. Studies have shown that changes in hormones could affect females’ food intake, binge eating, and emotional eating [43]. Secondly, females were more sensitive and concerned about their body image [44], thus exhibiting a propensity for weight management in their dietary behavior [45]. Finally, females were more vulnerable to external social and cultural pressures on their ideal body shape [37]. Many females engaged in social comparison regarding their bodies with peers, leading to potentially negative evaluations of their weight and physique [2]. Previous studies demonstrated that a high tendency towards appearance comparison was associated with eating disorders [46]. According to a Korean study, females may exert greater pressure on their appearance [47]. When they believe that their body does not meet social or cultural standards, they may try to change their body image by fasting. This finding emphasizes the importance of tailoring interventions specifically for female medical students to assist them in addressing the unique challenges associated with body image and intermittent fasting. Educational interventions should be designed to enhance medical students’ awareness of the risks related to extreme dietary habits. Students should be informed about the potential adverse physiological and psychological consequences of such behaviors. Workshops and seminars addressing body image issues and promoting balanced approaches to diet and exercise could be incorporated into the medical curriculum. The establishment of female-centered support groups and female advisors specializing in matters related to body image and dietary concerns should be prioritized to consider the overall well-being of students.

There was a stronger negative association between body image flexibility and intermittent fasting in first-year medical students. Firstly, first-year students may experience greater adaptation pressure from graduating from high school to entering university, which means adapting to new educational environments and social challenges [39]. Studies have shown that the adaptation process may harm the physical and mental health of college freshmen [48,49]. During this adaptation process, more and more scrutiny of their appearance may make them pay more attention to their body image, leading to a reduction in their dietary intake [50]. Secondly, for freshmen, the transition from high school to university is also a new stage of self-exploration and identity building [51,52]. They typically begin to contemplate their identity, interests, and values. Therefore, some first-year students may attempt to enhance their self-confidence and shape their image by improving their physical appearance, establishing social connections, and finding their place within the university [53]. Furthermore, first-year students may lack efficient stress and emotion management methods, resulting in an inability to properly alleviate stress and emotions during the transition period to university, raising the likelihood of intermittent fasting [54]. This finding serves as a reminder of the importance of prioritizing the physical and mental well-being of first-year college students to ensure a smooth transition during this period. Targeted interventions aimed at promoting the welfare of first-year students should be devised, encompassing strategies to address adaptation stress, enhance self-confidence, and cultivate effective stress management techniques. Further research should explore these facets in greater detail to formulate more comprehensive interventions tailored to accommodate the unique challenges faced by first-year medical college students.

We found that college students majoring in nursing and pharmacy had a stronger negative association with body image flexibility and intermittent fasting. This may be related to the characteristics of the different majors. Compared to doctors, nurses usually start to contact patients at the early stage of their training [55] and have more extensive and intimate interactions with patients during the whole nursing process [56,57]. Research indicated that possessing a healthy shape may enhance individuals’ body image and physical attractiveness [53]. Patients also exhibit a greater inclination to trust and respect nurses who present themselves with a tidy, healthy, and self-assured appearance [58]. Meanwhile, research suggested a strong association between self-esteem and body image [59]. Possessing a positive body image can enhance nurses’ self-esteem and self-confidence [60]. Such self-esteem and self-confidence may convey their dedication and professional competence, establish a sense of trust with patients, and facilitate effective communication and patient satisfaction. Similar to the nursing major, graduates of pharmacy programs often enter an industry that necessitates extensive interpersonal engagement. For instance, pharmacists are required to communicate extensively with physicians, nurses, and patients to enhance medication adherence and provide medication dispensing and counseling services [61]. A favorable professional image may contribute to their career advancement. This finding highlights the necessity for tailored interventions to cater to the unique requirements of students pursuing different academic majors within medical education institutions. However, research on different medical majors remains relatively restricted. Hence, this result encourages further research to delve more comprehensively into the potential mechanisms underpinning the identified correlations and to explore potential intervention strategies. By conducting broader investigations into the body image and dietary behaviors of medical school students, a wealth of information can be gathered to better inform the development of comprehensive well-being programs, ultimately leading to enhanced overall physical and mental health among future medical college students.

There was a stronger negative association between body image flexibility and intermittent fasting in medical students with high monthly living expenses. Students with higher living expenses usually come from affluent families. Studies have shown that family income is significantly related to nutritional intake [62]. Affluent individuals were more likely to adopt a high-quality diet and realize the importance of balanced nutrition to maintain health [63]. Therefore, students with high monthly living expenses have more economic resources at their disposal to obtain a healthy diet and exercise regularly and are naturally more inclined to maintain a healthy weight and body shape [64]. Furthermore, students with high monthly living expenses may be more likely to have access to good family education and mental health education [65], which can help them develop a positive body image and ultimately reduce the occurrence of intermittent fasting. Understanding the affluent socioeconomic background of students with high monthly living expenses can facilitate the development of health promotion strategies that are tailored to their specific needs and characteristics, enhance their physical image flexibility, and reduce unhealthy dietary behaviors. This finding emphasizes the potential role of socioeconomic factors in shaping medical students’ body image flexibility and dietary behavior. It also advocates for the development of strategic approaches aimed at mitigating socioeconomic disparities among medical students. By actively pursuing equitable resource allocation and equal opportunities, medical education institutions can work towards providing comprehensive physical and mental health education to all students, regardless of their economic backgrounds [66]. These strategies may encompass targeted educational initiatives, counseling services, and interventions designed to foster a positive body image. By recognizing the influence of social and economic background on students’ health-related choices, we can strive to create an inclusive environment that promotes physical and mental health and ensures students’ physical and mental well-being.

However, there were also limitations to our study. First, although the sample size of this study was 5138, it only represented all the medical students from one college in China. Therefore, the generalizability and extrapolation of our results to college students require further verification. Future research should consider employing more extensive multicenter samples that encompass medical students from varied geographical regions and cultural backgrounds to enhance the representativeness of the study results. Second, we only investigated whether there was intermittent fasting in the past year, but did not involve the duration and frequency of intermittent fasting. Future research could delve into the temporal aspects, encompassing both the duration and frequency of intermittent fasting behaviors to attain a more nuanced comprehension of this phenomenon. Third, this study relied on self-reported data to assess the association between intermittent fasting and body image flexibility, which might suffer from recall bias and desirability bias. Future research should contemplate the utilization of objective measurement tools to validate research findings, thereby promoting result precision. Fourthly, although we calculated E-values to evaluate unmeasured confounding, the potential presence of other unmeasured confounding variables cannot be entirely discounted. Future research should consider these unmeasured confounding variables for more robust results regarding the association between body image flexibility and intermittent fasting. Finally, we were unable to draw causal associations between body image flexibility and intermittent fasting from the cross-sectional study. Therefore, future research should prioritize longitudinal studies to enhance the understanding of the causal association between body image flexibility and intermittent fasting.

## 5. Conclusions

In this study, we found an association between body image flexibility and intermittent fasting among Chinese medical students. Individuals with higher levels of body image flexibility were less inclined to engage in intermittent fasting behaviors, particularly among female students, those majoring in pharmacy and nursing, first-year students, and those with higher living expenses. This study provides valuable insights and directions for the development of specific health intervention strategies aimed at enhancing the overall well-being of medical students. Firstly, personalized health intervention plans should be devised, offering tailored psychological support and health guidance based on students’ body image flexibility, thereby assisting them in cultivating healthier lifestyles. Secondly, a particular focus should be directed toward the female medical student population, implementing gender-specific intervention measures to mitigate unhealthy dietary habits. Furthermore, students in pharmacy and nursing majors appear to be more susceptible to engaging in intermittent fasting behaviors, indicating the need for specialized health education and support for students in these professions to ensure their physical and mental well-being. Lastly, consideration should be given to providing financial assistance to alleviate the negative impact of economic stressors on body image. These recommendations serve as a foundation for the formulation of targeted health interventions in the context of medical education, with the ultimate goal of improving the holistic health of medical students.

## Figures and Tables

**Table 1:** Characteristics of participants according to the median body image flexibility (*n* = 5138).

Variable	Total	Body Image Flexibility		t/Z/*χ^2^*	*p*-Value
		≤25.24 (*n* = 2852)	>25.24 (*n* = 2286)		
Age (years)	20.54 ± 1.62	20.58 ± 1.57	20.48 ± 1.69	2.18	0.030
BMI (kg/m^2^)	22.23 (19.23, 23.88)	21.97 (19.82, 24.69)	20.40 (18.68, 22.64)	−15.24	<0.001
Social media usage	48.91 ± 18.97	48.21 ± 18.33	49.80 ± 19.71	−2.99	0.003
Family health	38.00 ± 6.82	36.51 ± 6.09	39.86 ± 7.23	−17.99	<0.001
Gender, *n* (%)				0.05	0.829
Male	2119 (41.24)	1180 (41.37)	939 (41.08)		
Female	3019 (58.76)	1672 (58.63)	1347 (58.92)		
Ethnicity, *n* (%)				0.01	0.909
Han	4886 (95.10)	2713 (95.13)	2173 (95.06)		
Minority	252 (4.90)	139 (4.87)	113 (4.94)		
Academic year, *n* (%)				49.80	<0.001
Fifth	830 (16.15)	460 (16.13)	370 (16.19)		
Fourth	1249 (24.31)	745 (26.12)	504 (22.05)		
Third	1244 (24.21)	709 (24.86)	535 (23.40)		
Second	907 (17.65)	526 (18.44)	381 (16.67)		
First	908 (17.67)	412 (14.45)	496 (21.70)		
Major, *n* (%)				28.72	<0.001
Clinical medicine	862 (16.78)	1312 (46.00)	1108 (48.47)		
Oral medicine	625 (12.16)	350 (12.27)	275 (12.03)		
Medical imaging	2420 (47.10)	133 (4.66)	100 (4.37)		
Traditional Chinese medicine	289 (5.62)	378 (13.25)	331 (14.48)		
Pharmacy	233 (4.53)	138 (4.84)	151 (6.61)		
Nursing	709 (13.80)	541 (18.97)	321 (14.04)		
Hukou, *n* (%)				1.80	0.180
Non-agricultural	2031 (39.53)	1104 (38.71)	927 (40.55)		
Agricultural	3107 (60.47)	1748 (61.29)	1359 (59.45)		
Place of residence, *n* (%)				1.63	0.201
Urban	2781 (54.13)	1521 (53.33)	1260 (55.12)		
Rural	2357 (45.87)	1331 (46.67)	1026 (44.88)		
Monthly living expenses (CNY), n (%)				1.02	0.600
≤800	557 (10.84)	307 (10.76)	250 (10.94)		
801–1500	2981 (58.02)	1672 (58.63)	1309 (57.26)		
>1500	1600(31.14)	873 (30.61)	727 (31.80)		
Love experience, *n* (%)				5.11	0.078
Never been in love	2374 (46.20)	1280 (44.88)	1094 (47.86)		
Have been in love	1454 (28.30)	837 (29.35)	617 (26.99)		
Are in love	1310 (25.50)	735 (25.77)	575 (25.15)		
Intermittent fasting, *n* (%)				212.33	<0.001
No	3809(74.13)	1887 (66.16)	1922 (84.08)		
Yes	1329(25.87)	965 (33.84)	364 (15.92)		

Note: Continuous variables were presented as means ± standard deviations or the median and interquartile range, and categorical variables were presented as numbers and percentages. Percentages might not add up to 100% due to rounding. BMI indicates body mass index. CNY indicates the Chinese Yuan.

**Table 2:** Association between body image flexibility and intermittent fasting.

Model	OR (95%CI)	*p*
Model Ⅰ	0.93 (0.92, 0.94)	<0.001
Model Ⅱ	0.93 (0.92, 0.94)	<0.001
Model Ⅲ	0.94 (0.93, 0.95)	<0.001

Note: Model I was unadjusted. Model II adjusted for age and gender. Model III adjusted for age, BMI, social media usage, family health, gender, academic year, major, monthly living expenses, and love experience. OR: odds ratio. 95%CI: 95% confidence interval.

## Data Availability

The data in this study can be obtained from the corresponding author on reasonable request.

## Supplementary Materials

The following supporting information can be downloaded at: https://www.mdpi.com/article/10.3390/nu15194273/s1, Table S1: Factors associated with intermittent fasting.
