# Supplementary material for: Association between Body Image Flexibility and Intermittent Fasting in Chinese Medical Students: A Cross-Sectional Study
Source: Nutrients. 2023 Oct 6;15(19):4273. doi: 10.3390/nu15194273 (PMC10574058; doi:10.3390/nu15194273)
Supplement: Supplementary file 1 [file nutrients-15-04273-s001.zip › nutrients-2615922-supplementary.pdf]

Supplementary

Table S1 Factors associated with intermittent fasting

Variable	Intermittent fasting No (n=3809)	Intermittent fasting Yes (n=1329)	OR(95%CI)	P-value
Body image flexibility	26.23 ± 6.85	22.40 ± 7.45	0.93 (0.92, 0.94)	<0.001
Age (years)	20.47 ± 1.63	20.73 ± 1.58	1.11 (1.06, 1.15)	<0.001
BMI (kg/m^2)	21.11(19.05,23.62)	21.72(19.72,24.69)	1.04 (1.03, 1.06)	<0.001
Social media usage	49.25 ± 18.87	47.93 ± 19.23	1.00 (0.99, 1.00)	0.029
Family health	38.91 ± 6.53	35.39 ± 6.99	0.93 (0.92, 0.93)	<0.001
Gender, n (%)				
Male	1570 (41.22)	549 (41.31)	1.00	
Female	2239 (58.78)	780 (58.69)	1.00 (0.88, 1.13)	0.954
Ethnicity, n (%)				
Han	3635 (95.43)	1251 (94.13)	1.00	
Minority	174 (4.57)	78 (5.87)	1.30 (0.99, 1.71)	0.059
Academic year, n (%)				
Fifth	611 (16.04)	219 (16.48)	1.00	
Fourth	897 (23.55)	352 (26.49)	1.10 (0.90, 1.33)	0.369
Third	862 (22.63)	382 (28.74)	1.24 (1.02, 1.50)	0.034
Second	665 (17.46)	242 (18.21)	1.02 (0.82, 1.26)	0.889
First	774 (20.32)	134 (10.08)	0.48 (0.38, 0.61)	<0.001
Major, n (%)				
Clinical medicine	1797 (47.18)	623 (46.88)	1.00	
Oral medicine	441 (11.58)	184 (13.84)	1.20(0.99,1.46)	0.062
Medical imaging	176 (4.62)	57 (4.29)	0.93(0.68,1.28)	0.669
Traditional Chinese medicine	511 (13.42)	198 (14.90)	1.12(0.93,1.35)	0.245
Pharmacy	234 (6.14)	55 (4.14)	0.68(0.50,0.92)	0.013
Nursing	650 (17.06)	212 (15.95)	0.94(0.79,1.13)	0.506
Hukou, n (%)				
Non-agricultural	1479 (38.83)	552 (41.53)	1.00	
Agricultural	2330 (61.17)	777 (58.47)	0.89 (0.79, 1.02)	0.082
Place of residence, n (%)				
Urban	2033 (53.37)	748 (56.28)	1.00	
Rural	1776 (46.63)	581 (43.72)	0.89 (0.78, 1.01)	0.067
Monthly living expenses (CNY), n (%)				
≤800	393 (10.32)	164 (12.34)	1.00	
801-1500	2262 (59.39)	719 (54.10)	0.76(0.62,0.93)	0.008
＞1500	1154 (30.30)	446 (33.56)	0.93(0,75,1.15)	0.479
Love experience, n (%)				
Never been in love	1825 (47.91)	549 (41.31)	1.00	
Have been in love	1043 (27.38)	411 (30.93)	1.31(1.13,1.52)	<0.001

Are in love	941 (24.70)	369 (27.77)	1.30(1.12,1.52)	0.001

Note: Continuous variables are presented as means ± standard deviations or the median and interquartile range, and categorical variables are presented as numbers and percentages. Percentages may not add up to 100% due to rounding. BMI indicates body mass index. CNY indicates the Chinese Yuan. OR: odds ratio. 95%CI: 95% confidence interval.
